# A review on the role of gamma-butyrobetaine hydroxylase 1 antisense RNA 1 in the carcinogenesis and tumor progression

**DOI:** 10.1186/s12935-023-03113-3

**Published:** 2023-11-05

**Authors:** Juan Hu, Jipeng Liu, Siwei Zhou, Hongliang Luo

**Affiliations:** 1 Medical Service Division, The Second Affiliated Hospital of Nanchang University, Nanchang, 330000 Jiangxi China; 2 Department of Gastrointestinal Surgery, The Second Affiliated Hospital of Nanchang University, 1 Minde Road, Nanchang, 330000 Jiangxi People’s Republic of China; 3 Second School of Clinical Medicine, Nanchang University, Nanchang, 330038 Jiangxi China

**Keywords:** BBOX1-AS1, Tumors, Predictive biomarker, CeRNA network, Signaling pathway

## Abstract

Gamma-butyrobetaine hydroxylase 1 antisense RNA 1 (BBOX1-AS1), located on human chromosome 11 p14, emerges as a critical player in tumorigenesis with diverse oncogenic effects. Aberrant expression of BBOX1-AS1 intricately regulates various cellular processes, including cell growth, epithelial–mesenchymal transition, migration, invasion, metastasis, cell death, and stemness. Notably, the expression of BBOX1-AS1 was significantly correlated with clinical-pathological characteristics and tumor prognoses, and it could also be used for the diagnosis of lung and esophageal cancers. Through its involvement in the ceRNA network, BBOX1-AS1 competitively binds to eight miRNAs in ten different cancer types. Additionally, BBOX1-AS1 can directly modulate downstream protein-coding genes or act as an mRNA stabilizer. The implications of BBOX1-AS1 extend to critical signaling pathways, including Hedgehog, Wnt/β-catenin, and MELK/FAK pathways. Moreover, it influences drug resistance in hepatocellular carcinoma. The present study provides a systematic review of the clinical significance of BBOX1-AS1’s aberrant expression in diverse tumor types. It sheds light on the intricate molecular mechanisms through which BBOX1-AS1 influences cancer initiation and progression and outlines potential avenues for future research in this field.

## Introduction

Long non-coding RNAs (lncRNAs) are RNA molecules exceeding 200 nucleotides in length, devoid of protein-coding ability [1–4]. Once considered “RNA junk,” they have emerged as rising stars, garnering increasing attention as attractive targets in human diseases [5–8], particularly in cancers [9–12]. The lncRNAs can be broadly classified based on their relationship with neighboring protein-coding genes, falling into categories such as sense, antisense, intronic, bidirectional, or intergenic [13, 14]. The functions of lncRNAs are significantly influenced by their subcellular localization [1, 15]. In the nucleus, they participate in regulating gene expression at epigenetic and transcriptional levels. Conversely, in the cytoplasm, lncRNAs interact with proteins and modulate mRNA metabolism. In cancer, lncRNAs play an important role in the ceRNA network [16–19]. By acting as “sponges” for microRNAs (miRNAs) and sharing miRNA response elements (MREs) with target mRNAs, lncRNAs competitively bind to miRNAs, influencing miRNA availability and indirectly regulating the expression of target genes [20]. Dysregulation of the ceRNA network involving lncRNAs has been observed in various cancer types, where some lncRNAs act as oncogenic ceRNAs [21–24], while others function as tumor suppressor ceRNAs [25–28]. The growing body of evidence underscores the critical role of lncRNAs as essential modulators of diverse biological processes relevant to tumorigenesis and cancer progression [29–31]. Their intricate involvement in the ceRNA network further highlights their significance in cancer biology, offering promising avenues for potential therapeutic interventions.

In humans, Gamma-butyrobetaine hydroxylase 1 (BBOX1) antisense 1 (BBOX1-AS1) is classified as an lncRNA gene. It is located on human chromosome 11 at position p14.2-p14.1. Comprising 7 exons, this gene spans a length of 172,928 nucleotides (nt) (https://www.ncbi.nlm.nih.gov/gene/103695435). The lncRNA produced by the BBOX1-AS1 gene exhibits five distinct splice variants, namely ENST00000525302.5, ENST00000530430.1, ENST00000531363.1, ENST00000526061.5, and ENST00000670273.1, each varying in size from 471 base pairs (bp) for ENST00000526061.5 to 879 bp for ENST00000530430.1 (http://www.ensembl.org/Homo_sapiens/Gene/Summary?db=core;g=ENSG00000254560;r=11:27047186-27220113).

**Fig. 1:**
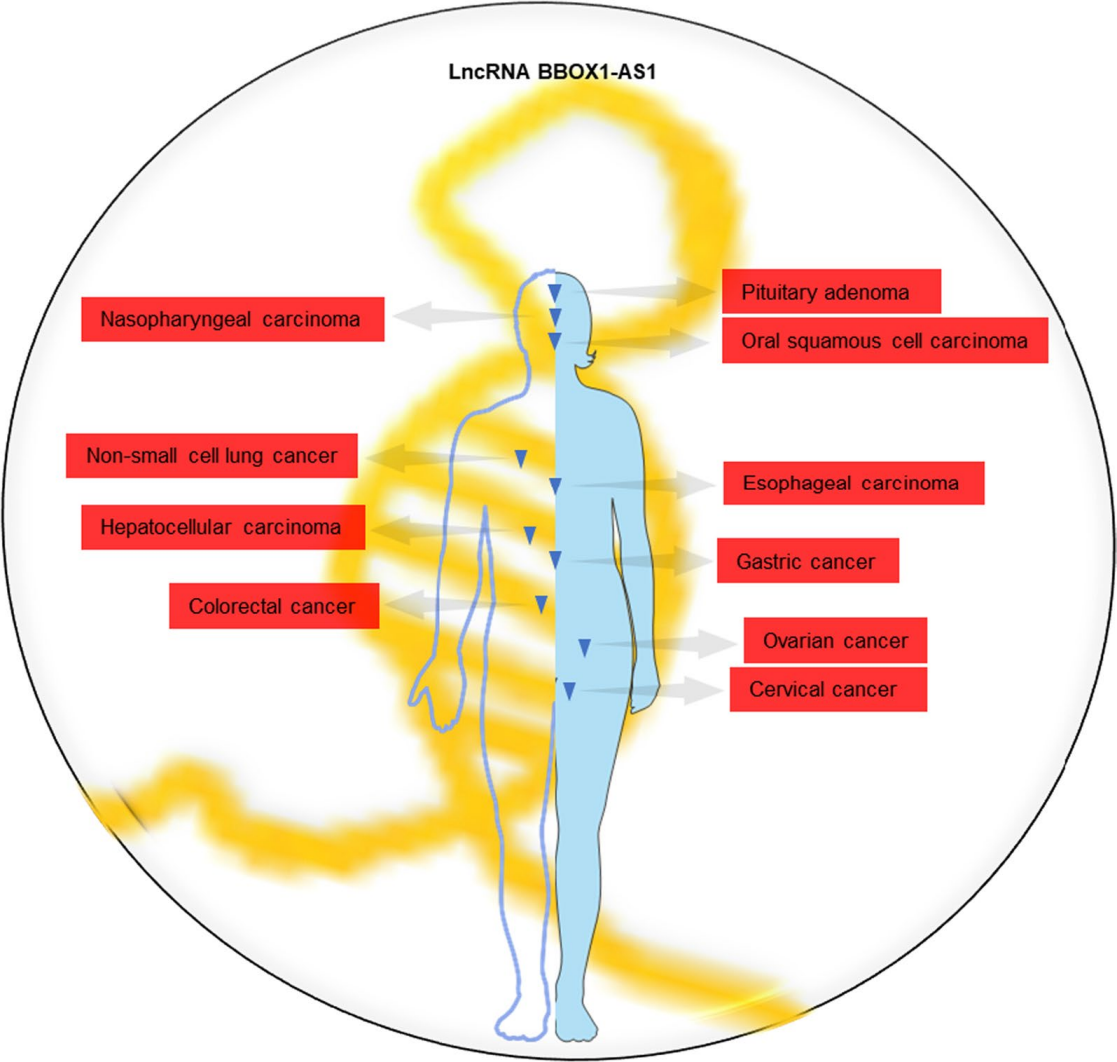
LncRNA BBOX1-AS1 is highly expressed and studied in various human cancers

LncRNA BBOX1-AS1 has recently emerged as a key player in the pathogenesis of various diseases, including recurrent pregnancy loss [32] and premature ovarian failure [33]. Remarkably, its involvement in cancer progression has garnered growing interest. BBOX1-AS1 demonstrates upregulation in a wide range of human tumors (Fig. 1), including pituitary adenoma (PA) [34], oral squamous cell carcinoma (OSCC) [35], nasopharyngeal carcinoma (NPC) [36, 37], non-small cell lung cancer (NSCLC) [38–40], esophageal carcinoma (EC) [41–45], hepatocellular carcinoma (HCC) [46, 47], gastric cancer (GC) [48], colorectal cancer (CRC) [49, 50], ovarian cancer (OC) [51], and cervical cancer (CC) [52, 53]. High expression levels of BBOX1-AS1 in tumor patients have been linked to adverse clinicopathological features and poor prognosis, including lymph node metastasis, tumor size, clinical stage, overall survival (OS), and disease-free survival (DFS). Furthermore, BBOX1-AS1 plays a crucial role in vital biological processes, such as promoting tumor cell growth and invasion while inhibiting cell apoptosis. Considering its pivotal role in tumor progression, BBOX1-AS1 is anticipated to serve as a valuable biomarker for the diagnosis, prognosis, and development of effective therapeutic strategies across a range of malignancies.

In this review, we present a comprehensive summary of the most recent research progress regarding the roles played by BBOX1-AS1 in tumor development. We focus on BBOX1-AS1 expression patterns, associated clinical characteristics, its potential as a prognostic and diagnostic marker, and its biological functions in tumor development. Additionally, we delve into the underlying mechanisms driving BBOX1-AS1’s effects and explore its potential clinical applications in the context of different malignancies. This review sheds light on the promising prospects of BBOX1-AS1 as a target for therapeutic interventions in various cancer types.

## BBOX1-AS1 expression and its associations with clinical features in human tumors

As a recently identified oncogene, BBOX1-AS1 exhibits significant upregulation in various types of cancerous samples originating from the human nervous, respiratory, digestive, and reproductive systems, as detailed in Table 1. Notably, Ensembl annotates five splice variants of BBOX1-AS1. We also listed the qRT-PCR primers used in each study and the specific variants of lncRNA BBOX1-AS1 detected (Table 2). It is worth noting that the studies conducted across these tumor types did not analyze the same variants.

Research has investigated the relationship between BBOX1-AS1 expression and clinicopathological features in these cancer types (Table 1). In respiratory system tumors, BBOX1-AS1 exhibits a significant positive correlation with larger tumor size, lymph node metastasis, and advanced Tumour-Node-Metastasis (TNM) stage in NSCLC [39, 40]. Similarly, in digestive system tumors [41, 43, 44, 46–48], BBOX1-AS1 shows a significant positive association with larger tumor size, lymph node metastasis, or advanced TNM stage. Notably, high BBOX1-AS1 expression is positively correlated with lymph node metastasis in esophageal carcinoma [41, 44] and gastric cancer [48]. Elevated BBOX1-AS1 expression is linked to positive vascular invasion, higher tumor grade, and advanced clinical stage in hepatocellular carcinoma [46, 47]. In the context of reproductive tumors, high expression of BBOX1-AS1 indicates more aggressive phenotypes [53], including larger tumor size, poor differentiation, distant metastasis, and higher FIGO stage in cervical cancer.

**Table 1:** Expression of BBOX1-AS1 and its associations with clinical features, prognosis, and diagnosis in human tumor samples

Tumor type	Expression			Significant clinical features	Prognosis	Diagnosis	Ref.
	Tumor vs. normal tissues	Fold change	Sample size				
Pituitary adenoma	Upregulated	~ 6.5-fold	38 pairs of adenoma and adjacent normal tissues (qRT-PCR)	–	–	–	[34]
Oral squamous cell carcinoma	Upregulated	~ 4-fold	50 pairs of adenoma and adjacent normal tissues (qRT-PCR)	–	–	–	[35]
Nasopharyngeal carcinoma	Upregulated	~ 2.5-fold	45 pairs of adenoma and adjacent normal tissues (qRT-PCR)	–	–	–	[37]
Nasopharyngeal carcinoma	Upregulated	~ 6-fold	45 paired of adenoma and adjacent normal tissues (qRT-PCR)	–	–	–	[36]
Non-small cell lung cancer	Upregulated	~ 1.5-fold	135 pairs of adenoma and adjacent normal tissues (qRT-PCR)	Lymph node metastasis, TNM stage	OS	–	[40]
Non-small cell lung cancer	Upregulated	~ 2-fold	76 pairs of adenoma and adjacent normal tissues (qRT-PCR)	Tumor size, lymph node metastasis, TNM stage	OS	–	[39]
		–	526 cancer and 59 normal samples in LUAD, and 501 cancer and 49 normal samples in LUSC (starBase v3.0 database)				
		–	486 cancer and 338 normal samples in LUSC (GEPIA database)				
		–	GEO datasets (GSE18842 and GSE19188)				
Lung squamous cell carcinoma	Upregulated	–	3 pairs of LUSC tissues and normal lung tissues (qRT-PCR)	Ethnicity	–	AUC of 0.983 (95% CI 0.973–0.993, P < 0.0001)	[38]
		–	502 cancer and 49 normal samples in LUSC (TCGA dataset)				
		–	GEO datasets (GSE19188, GSE30219, GSE103512, E‑MTAB‑5231)				
Esophageal carcinoma	Upregulated	~ 12-fold	45 pairs of adenoma and adjacent normal tissues (qRT-PCR)	–	–	–	[45]
Esophageal squamous cell carcinoma	Upregulated	~ 2-fold	78 pairs of adenoma and adjacent normal tissues (qRT-PCR)	Tumor size, lymph node metastasis, TNM stage	OS		[44]
		–	182 adenoma and 286 normal tissues (GEPIA database)				
Esophageal squamous cell carcinoma	Upregulated	~ 2.75-fold	45 pairs of adenoma and adjacent normal tissues (qRT-PCR)	Tumor size	OS	AUC of 0.7668 (95% CI 0.6399 to 0.8937, P < 0.001)	[43]
		–	182 adenoma and 13 normal tissues (GEPIA database)				
Esophageal squamous cell carcinoma	Upregulated	–	182 adenoma and 286 normal tissues (GEPIA database)	–	–	–	[42]
		–	162 cancer and 11 normal samples (starBase v3.0 database)				
Esophageal carcinoma	Upregulated	~ 3.75-fold	45 pairs of adenoma and adjacent normal tissues (qRT-PCR)	Lymph node metastasis, TNM stage	OS	–	[41]
Gastric cancer	Upregulated	~ 3-fold	40 pairs of adenoma and adjacent normal tissues (qRT-PCR)	Lymph node metastasis	–	–	[48]
		–	408 tumor samples and 211 normal samples (GEPIA database)				
Hepatocellular carcinoma	Upregulated	~ 2-fold	83 pairs of adenoma and adjacent normal tissues (qRT-PCR)	Vascular invasion, TNM stage	OS	–	[46]
Hepatocellular carcinoma	Upregulated	~ 2-fold	374 tumor samples and 50 normal samples (GENCODE database)	Vascular invasion, tumor grade, tumor stage	OS, DFS		[47]
Ovarian cancer	Upregulated	–	426 tumor samples and 88 normal samples (TCGA database)	–	–	–	[51]
Cervical cancer	Upregulated	~ 2-fold	100 pairs of adenoma and adjacent normal tissues (qRT-PCR)	Tumor size, differentiation, distant metastasis, FIGO stage	OS	–	[53]
		–	306 tumor samples and 13 normal samples (GEPIA database)				
Cervical cancer	Upregulated	–	306 tumor samples and 13 normal samples (GEPIA database)	–	–	–	[52]

*qRT-PCR* quantitative reverse transcription polymerase chain reaction, *LUAD* lung adenocarcinoma, *LUSC* lung squamous cell carcinoma, *OS* overall survival, *DFS* disease-free survival

“–”: indicates missing or not applicable data

**Table 2:** qRT-PCR primers used in each study and lncRNA BBOX1-AS1 variants detected in tissue samples

Tumor type	qRT-PCR primers	LncRNA BBOX1-AS1 variants	Ref.
Pituitary adenoma	–	–	[34]
Oral squamous cell carcinoma	Forward: 5′-TGTGTGTTTCCTGAGGCCTC-3′ Reverse: 5′-CGCCTCTCTTGGAACACCTT-3′	ENST00000526061.5	[35]
Nasopharyngeal carcinoma	Forward: 5ʹ-TGTGTGTTTCCTGAGGCCTC-3′ Reverse: 5ʹ-CGCCTCTCTTGGAACACCTT-3′	ENST00000526061.5	[37]
Nasopharyngeal carcinoma	Forward: 5′-TGCAACTCCAAACCTAACGC-3′ Reverse: 5′-GAGTGACTGGGGTCAGGGTA-3′	ENST00000525302.5	[36]
Non-small cell lung cancer	–	–	[40]
Non-small cell lung cancer	Forward: 5′-CAGACTCCTGCTTTGCTCTT-3′ Reverse: 5′-GGAAGCATCTTCTCAGCTTCT-3′	ENST00000530430.1	[39]
Lung squamous cell carcinoma	Forward: 5′‑GATGGGCACATTTGGAAGTT‑3′ Reverse: 5′‑CAGCGTTAGGTTTGGAGTTG‑3′	ENST00000525302.5 ENST00000530430.1	[38]
Esophageal carcinoma	Forward: 5′-CCG CTG ACA GGT CTA GGA GT-3′ Reverse: 5′-AGT GAC TGG GGT CAG GGT AA-3′	ENST00000525302.5	[45]
Esophageal squamous cell carcinoma	Forward: 5′- CAGACTCCTGCTTTGCTCTT-3′ Reverse: 5′- GGAAGCATCTTCTCAGCTTCT-3′	ENST00000526061.5 ENST00000525302.5 ENST00000530430.1	[44]
Esophageal squamous cell carcinoma	Forward: 5′- CGAGACTCCGTGGGCGTAGG-3′ Reverse: 5′- CGGGCGGCACCTGGAAAATC-3′	ENST00000531363.1 ENST00000530430.1	[43]
Esophageal squamous cell carcinoma	–	–	[42]
Esophageal carcinoma	Forward: 5′-CCGCTGACAGGTCTAGGAGT-3′ Reverse: 5′-AGTGACTGGGGTCAGGGTAA-3′	ENST00000525302.5	[41]
Gastric cancer	Forward: 5′-TGCAACTCCAAACCTAACG-3′ Reverse: 5′-GAGTGACTGGGGTCAGGGTA-3′	ENST00000525302.5	[48]
Hepatocellular carcinoma	Forward: 5′-CCTGAATACCAAAGAGGGCCG-3′ Reverse: 5′-TGAAGCCTCTCTCTGCTAGGT-3′	ENST00000525302.5, ENST00000530430.1, ENST00000531363.1, ENST00000526061.5, ENST00000670273.1	[46]
Hepatocellular carcinoma	Forward: 5′-CCTGAATACCAAAGAGGGCCG-3′ Reverse: 5′-TGAAGCCTCTCTCTGCTAGGT-3′	ENST00000525302.5, ENST00000530430.1, ENST00000531363.1, ENST00000526061.5, ENST00000670273.1	[47]
Ovarian cancer	–	–	[51]
Cervical cancer	–	–	[53]
Cervical cancer	–	–	[52]

*qRT-PCR* quantitative reverse transcription polymerase chain reaction

“–”: indicates missing or not applicable data

## BBOX1-AS1 as a diagnostic and prognostic marker in cancers

**Fig. 2:**
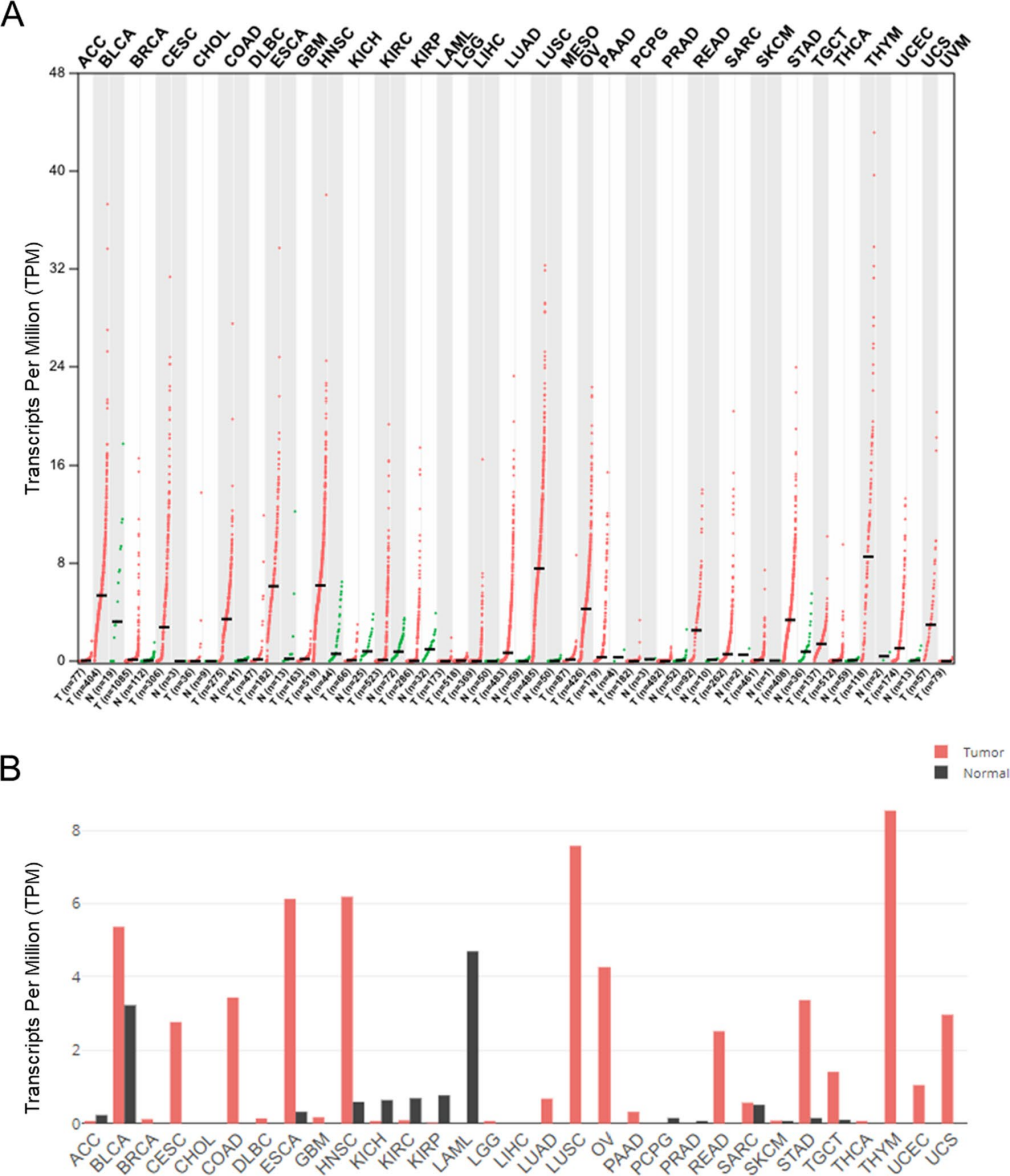
Gene expression of BBOX1-AS1 in 31 different tumor samples and normal tissues—dot plot (**A**) and bar plot (**B**)

As previously mentioned, numerous studies consistently demonstrate a significant upregulation of BBOX1-AS1 in tumor tissues when compared to their corresponding normal samples (Table 1). To comprehensively assess the expression pattern of BBOX1-AS1 in various cancers, we conducted an extensive analysis of its expression levels across 31 different tumor types. This analysis was performed using the GEPIA 2 web server (http://gepia2.cancer-pku.cn/#index) and is depicted in Fig. 2. The findings revealed that BBOX1-AS1 consistently exhibited elevated expression levels in the majority of cancerous lesions when contrasted with their corresponding normal tissues. Conversely, BBOX1-AS1 expression in most normal tissues remained relatively low. This significant dysregulation of BBOX1-AS1 in cancer tissues underscores its potential as a promising prognostic and diagnostic marker for various cancer types.

Several studies have reported a significant relationship between aberrant BBOX1-AS1 expression and cancer patient prognosis. As indicated in Table 1, high expression of BBOX1-AS1 was found to predict poor overall survival in four types of cancers: non-small cell lung cancer [39, 40], esophageal squamous cell carcinoma [41, 43, 44], hepatocellular carcinoma [46, 47], and cervical cancer [53], as well as inferior disease-free survival in hepatocellular carcinoma [47]. Additionally, several prognostic models that incorporate BBOX1-AS1 have been developed. For example, a two-lncRNA panel (BBOX1-AS1 and FOXP4-AS1) displayed moderate predictive accuracy in CRC [49], and an eight-lncRNA signature associated with vascular invasion was identified, showing strong predictive capability for clinical outcomes in HCC patients [47].

In addition, BBOX1-AS1 has demonstrated diagnostic significance in lung cancer [38] and esophageal carcinoma [43]. Receiver Operating Characteristic (ROC) analysis revealed that BBOX1-AS1 could effectively distinguish tumor tissues from normal tissues, achieving an AUC value of 0.983 in lung squamous cell carcinoma [38] and 0.7668 in esophageal squamous cell carcinoma [43]. However, the diagnostic potential of BBOX1-AS1 in other types of cancers remains unexplored. Exploring the diagnostic utility of BBOX1-AS1 in various cancer types presents a promising avenue for future research.

## The functions of BBOX1-AS1 in human tumors

**Fig. 3:**
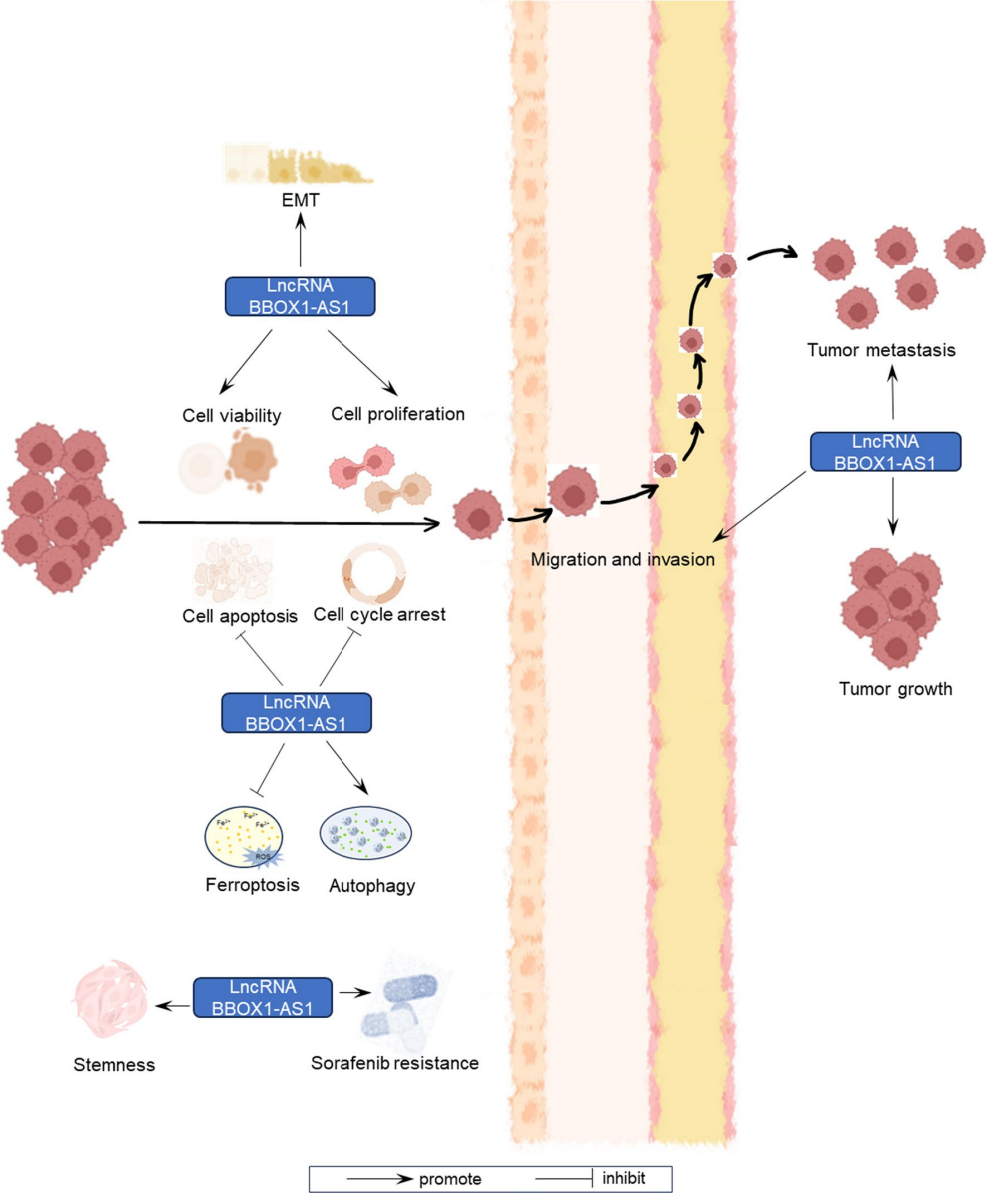
Effects of lncRNA BBOX1-AS1 on various tumor-related processes to regulate crucial aspects of cancer progression

Research into the involvement of BBOX1-AS1 in various cancers has been conducted through in vivo and in vitro experiments, as summarized in Table 3. The expression of BBOX1-AS1 is significantly higher in numerous tumor cell lines when compared to their corresponding normal cells. This lncRNA is found in both the cytoplasm and the nucleus, with its primary location reported to be in the cytoplasm for the majority studied tumor cell lines. BBOX1-AS1 plays an oncogenic role in tumor development (Fig. 3). Overexpression of BBOX1-AS1 leads to enhanced cell proliferation, cell viability, migration, invasion, epithelial-mesenchymal transition (EMT), stemness, autophagy, drug resistance, tumor growth, and metastasis. Additionally, BBOX1-AS1 suppresses apoptosis, ferroptosis, and cell cycle arrest in tumor cells. These findings underscore the significance of BBOX1-AS1 as a critical regulator in various aspects of tumor progression.

**Fig. 4:**
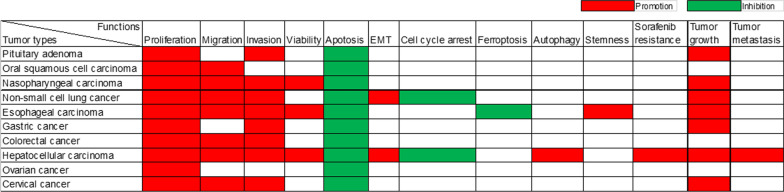
Diverse functions of lncRNA BBOX1-AS1 in ten different human tumors from in vivo and in vitro experiments

Upregulated BBOX1-AS1 promotes cell proliferation in 10 different types of tumors and inhibits cell apoptosis in 9 different types of tumors. Epithelial-mesenchymal transition (EMT) is a process that boosts cells’ capacity for invasion and metastasis [54, 55]. In non-small cell lung cancer and hepatocellular carcinoma, elevated levels of BBOX1-AS1 lead to the promotion of EMT. The migration and invasion ability of tumor cells decreases after BBOX1-AS1 knockdown in multiple tumors. BBOX1-AS1 also inhibits ferroptosis [44] and enhances stemness [42] in esophageal carcinoma, promotes autophagy and sorafenib resistance in hepatocellular carcinoma [46]. Knockdown of BBOX1-AS1 resulted in cell cycle arrest in lung squamous cell carcinoma [38] and hepatocellular carcinoma [47]. Furthermore, BBOX1-AS1 could promote tumor growth and metastasis. The functions of BBOX1-AS1 in different human tumors from publications are displayed in Fig. 4.

**Table 3:** The functions and regulatory mechanisms of BBOX1-AS1 in different human cancers

Tumor type	Expression		Experiments	Functions	Regulatory mechanism	Signaling pathway	Ref.
	Tumor vs. normal cell lines	Subcellular locations					
Pituitary adenoma	Upregulated	Mainly in the cytoplasm	In vitro/in vivo	Cell proliferation, invasion, apoptosis inhibition; tumor growth	BBOX1-AS1/miR-361-3p/E2F1	–	[34]
Oral squamous cell carcinoma	Upregulated	Mainly in the cytoplasm	In vitro	Cell proliferation, migration, apoptosis inhibition	BBOX1-AS1/miR-3940-3p/LAMC2	–	[35]
Nasopharyngeal carcinoma	Upregulated	Mainly in the cytoplasm	In vitro/in vivo	Cell viability, migration, apoptosis inhibition; tumor growth	BBOX1-AS1/miR-204-5p/MUC4	–	[36]
Nasopharyngeal carcinoma	Upregulated	–	In vitro	Cell proliferation, migration, invasion	BBOX1-AS1/miR-3940-3p/KPNA2	–	[37]
Non-small cell lung cancer	Upregulated	–	In vitro	Cell proliferation, migration, invasion	BBOX1-AS1/miR-361-3p	–	[40]
Non-small cell lung cancer	Upregulated	Predominantly in the cytoplasm	In vitro/in vivo	Cell proliferation, migration, invasion, EMT; tumor growth	BBOX1-AS1/miR-27a-5p/MELK	MELK/FAK signaling	[39]
Lung squamous cell carcinoma	Upregulated	Mainly in the cytoplasm	In vitro	Cell proliferation, migration, cell cycle arrest	–	–	[38]
Esophageal carcinoma	Upregulated	Mainly in the cytoplasm	In vitro/in vivo	Cell proliferation, apoptosis inhibition; tumor growth	BBOX1-AS1/miR-361-3p/COL5A1	–	[45]
Esophageal squamous cell carcinoma	Upregulated	–	In vitro/in vivo	Cell proliferation, invasion, migration, apoptosis inhibition, ferroptosis inhibition; tumor growth and proliferation	BBOX1-AS1/miR-513a-3p/SLC7A11	–	[44]
Esophageal squamous cell carcinoma		Mostly in the nucleus	In vitro	Cell proliferation, migration, invasion, apoptosis inhibition	HOXB7, β-catenin	Wnt/β-catenin pathway	[43]
Esophageal squamous cell carcinoma	Upregulated	Mainly distributed in the cytoplasm	In vitro	Cell proliferation, stemness	BBOX1-AS1/miR-506-5p/EIF5A/PTCH1	Hedgehog signaling pathway	[42]
Esophageal carcinoma	Upregulated	Principally in the cytoplasm	In vitro/in vivo	Cell proliferation, viability, migration, apoptosis; tumor growth and proliferation	BBOX1-AS1/miR-361-3p/COL1A1	–	[41]
Gastric cancer	Upregulated	Mainly in the cytoplasm	In vitro/in vivo	Cell proliferation, invasion, apoptosis inhibition; tumor growth	BBOX1-AS1/miR-361-3p/MUC13	–	[48]
Colorectal cancer	Upregulated	Mainly in the cytoplasm	In vitro	Cell proliferation, migration, invasion, apoptosis inhibition	BBOX1-AS1/miR-361-3p/SH2B1	–	[50]
Hepatocellular carcinoma	Upregulated	Predominantly in the cytoplasm	In vitro/in vivo	Cell proliferation, invasion, apoptosis inhibition, EMT, autophagy, cell viability, sorafenib resistance; tumor growth, proliferation, EMT, tumor metastasis	BBOX1-AS1/miR-361-3p/PHF8	–	[46]
Hepatocellular carcinoma	–	–	In vitro/in vivo	Cell proliferation, migration, invasion, cell cycle arrest; tumor growth	–	–	[47]
Ovarian cancer	Upregulated		In vitro	Cell proliferation, apoptosis inhibition	BBOX1-AS1/miR-361-3p/PODXL	–	[51]
Cervical cancer	Upregulated	Mainly in the cytoplasm	In vitro/in vivo	Cell proliferation, apoptosis inhibition, migration, invasion; tumor growth, proliferation, EMT	BBOX1-AS1/miR-361-3p/HOXC6/HuR	–	[53]
Cervical cancer	–	–	–	–	BBOX1-AS1-hsa-miR-125b-5p/hsa-miR-125a-5p-CDKN2A	–	[52]

*EMT* epithelial–mesenchymal transition

“–”: indicates missing or not applicable data

## BBOX1-AS1 modulates gene expression through a ceRNA network

The discovery of competing endogenous RNAs (ceRNAs) network has been on the rise. These ceRNAs act as protective shields for mRNAs against miRNA-mediated inhibition. Within the context of cancer, the ceRNA regulatory network orchestrated by lncRNAs assumes a vital role [18, 56–58].

**Fig. 5:**
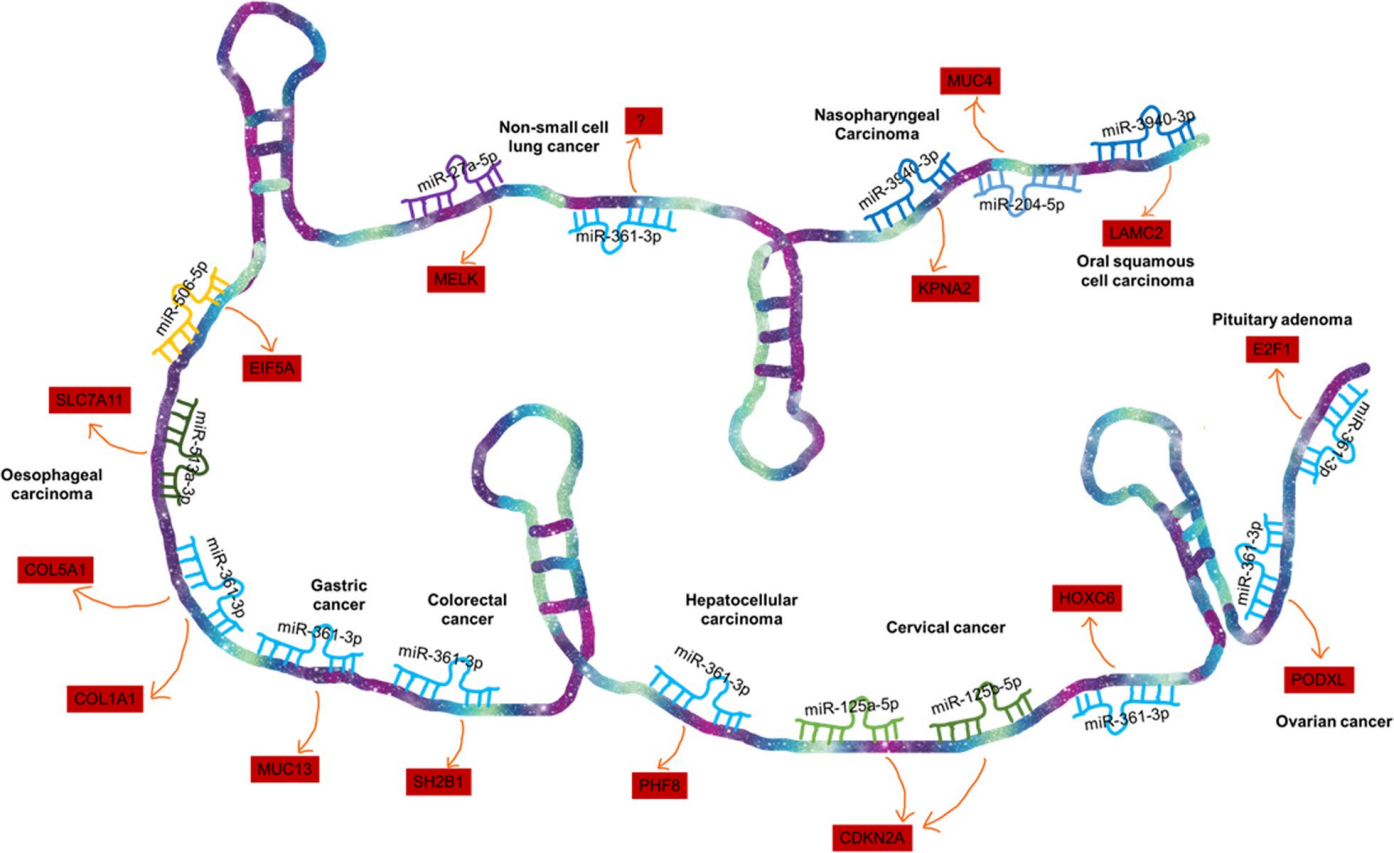
The ceRNA network of lncRNA BBOX1-AS1 in various human cancers

In the case of BBOX1-AS1, its ceRNA network involves eight miRNAs across ten different types of cancers (Fig. 5). These miRNAs include miR-3940-3p, miR-204-5p, miR-361-3p, miR-27a-5p, miR-506-5p, miR-513a-3p, miR-125a-5p, and miR-125b-5p.

BBOX1-AS1 exerts its regulatory function in different tumors by competitively binding to various miRNAs. For example, in oral squamous cell carcinoma [35], BBOX1-AS1 acts as a sponge for miR-3940-3p, resulting in the upregulation of LAMC2, which promotes tumor cell proliferation and migration while inhibiting apoptosis.

Interestingly, BBOX1-AS1 can also participate in the development of the same tumor by interacting with multiple miRNAs. For instance, in NPC, two different ceRNA mechanisms involving BBOX1-AS1 have been identified: BBOX1-AS1 can promote the proliferation, migration, and invasion of NPC cells by targeting miR-3940-3p/KPNA2 axis [37] and miR-204-5p/MUC4 axis [36]. Similarly, in esophageal carcinoma [41, 42, 44, 45], BBOX1-AS1 interacts with three miRNAs (miR-506-5p, miR-513a-3p, miR-361-3p) and subsequently upregulates the expression of four target genes (EIF5A, SLC7A11, COL5A1, COL1A1), promoting tumor cell growth, migration, invasion, and accelerating the progression and metastasis of esophageal cancer.

Furthermore, BBOX1-AS1 can participate in the progression of various tumors by acting on the same miRNA. BBOX1-AS1 competitively binds miR-361-3p, leading to the upregulation of E2F1 in pituitary adenoma [34], COL5A1 and COL1A1 in oesophageal carcinoma [41, 45], MUC13 in gastric cancer [48], SH2B1 in colorectal cancer [50], PHF8 in hepatocellular carcinoma [46], HOXC6 in cervical cancer [53], and PODXL in ovarian cancer [51], thereby promoting tumor cell proliferation, invasion and metastasis.

## BBOX1-AS1 regulates mRNA stability and downstream targets

**Fig. 6:**
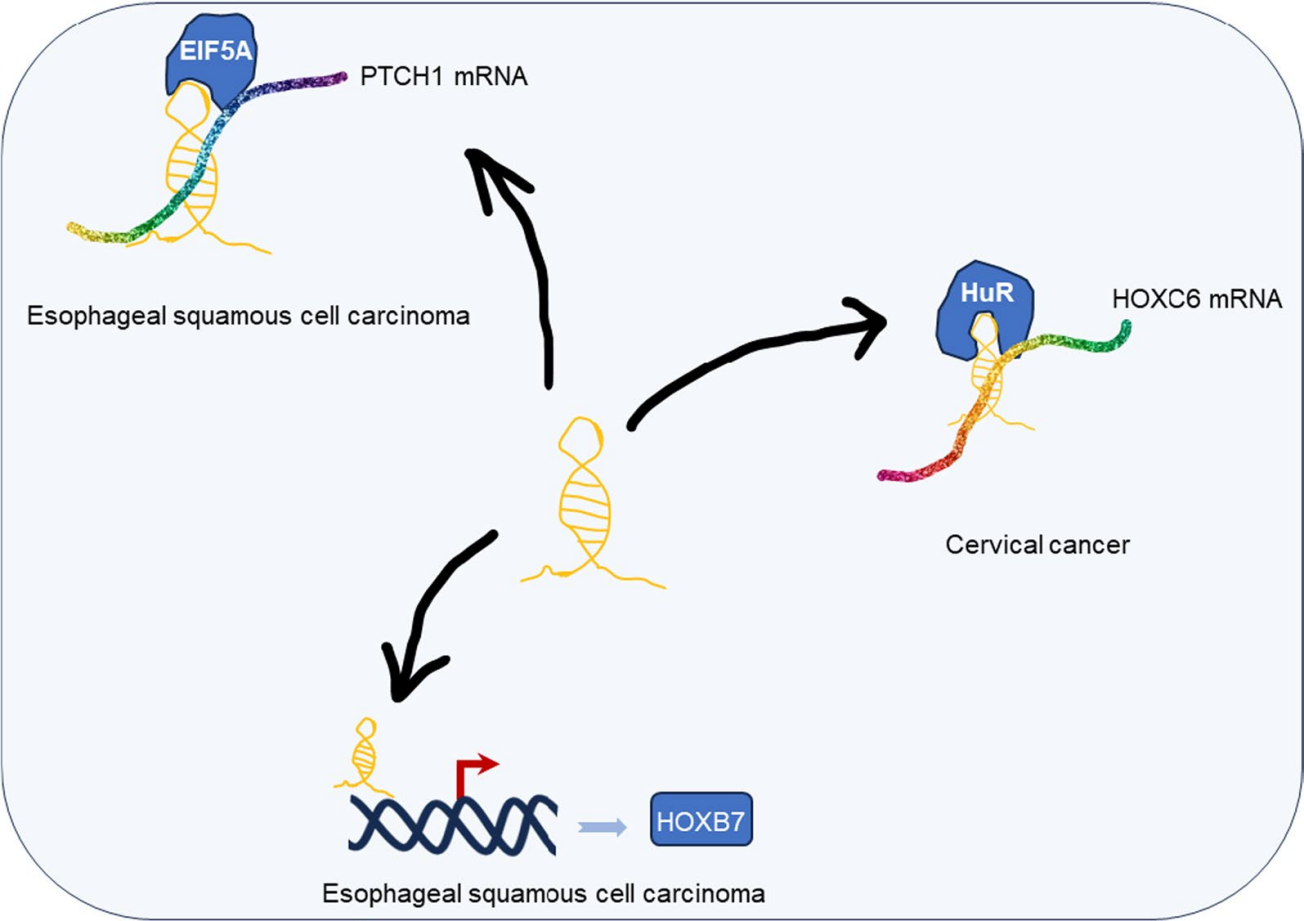
LncRNA BBOX1-AS1 functions as a direct regulator of downstream genes and an mRNA stabilizer in cervical cancer and esophageal squamous cell carcinoma

BBOX1-AS1, as a pivotal player in gene regulation, exerts its influence not only by modulating gene expression through the ceRNA network, as mentioned previously, but also by regulating mRNA stability and influencing downstream targets (Fig. 6).

In terms of mRNA stability, BBOX1-AS1 can interact with specific mRNAs and modulates their degradation or stabilization. By binding to certain mRNAs, BBOX1-AS1 can protect them from degradation, leading to increased stability of these mRNAs. This extended stability results in higher levels of the corresponding proteins encoded by these mRNAs, thereby impacting various cellular processes. For example, in esophageal squamous cell carcinoma, BBOX1-AS1 targets miR-506-5p/EIF5A to maintain PTCH1 mRNA stability, leading to increased PTCH1 expression and enhanced cancer development [42]. Similarly, in cervical cancer, BBOX1-AS1 can interact with HuR, enhancing the mRNA stability of HOXC6, subsequently upregulating the HOXC6 protein expression, and promoting cancer cell proliferation, migration, and invasion abilities [53].

Furthermore, BBOX1-AS1’s influence extends beyond the ceRNA network, as it can also impact downstream targets. For example, HOXB7 is an essential downstream target of BBOX1-AS1, and HOXB7 is involved in the activation of the Wnt/β-catenin signaling pathway, contributing to malignant phenotypes in esophageal squamous cell carcinoma [43].

The combined regulatory mechanisms involving ceRNA and mRNA stability modulation make BBOX1-AS1 a multifaceted regulator of gene expression. Its dysregulation in cancer highlights its importance as a potential therapeutic target and emphasizes the need for further investigation into its precise functions and molecular interactions.

## BBOX1-AS1 influences the signaling pathways

**Fig. 7:**
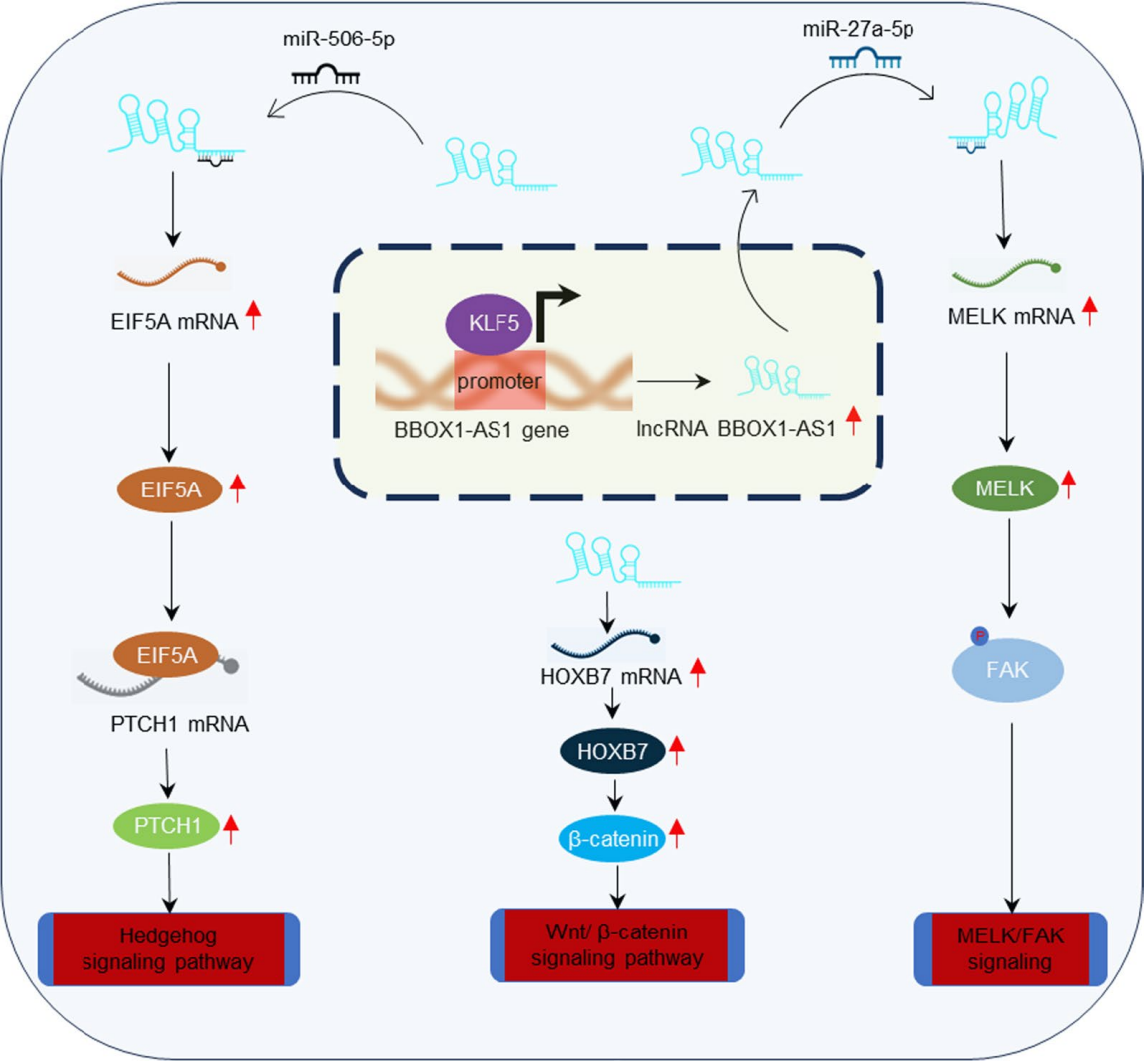
Signaling pathways mediated by lncRNA BBOX1-AS1 in tumorigenesis

Accumulating evidence suggests that lncRNAs play a significant role in regulating multiple signaling pathways, offering insights into the development of targeted therapies [58–63]. Currently, BBOX1-AS1 has been confirmed to participate in the regulation of the Hedgehog signaling pathway [42]. And the genes regulated by BBOX1-AS1 are also associated with other cancer-related signaling pathways, including the Wnt/β-catenin signaling pathway [43] and MELK/FAK signaling [39]. BBOX1-AS1’s involvement with these signaling pathways implies its broader impact on cancer cell behavior and therapeutic responses (Fig. 7).

The Hedgehog pathway is a fundamental cellular signaling pathway involved in embryonic development [64, 65] and tissue homeostasis [66, 67]. Dysregulation of this pathway also has been linked to the development and progression of various cancers [68, 69]. BBOX1-AS1 has been shown to upregulate PTCH1 by sponging miR-506-5p, which in turn upregulates EIF5A, stabilizing PTCH1 mRNA and ultimately activating the Hedgehog signaling pathway [42]. By modulating this critical signaling pathway, BBOX1-AS1 can impact ESCC cell proliferation and stemness, both crucial processes involved in cancer development [70–72].

The Wnt pathway is essential for tissue development and cell fate determination [73, 74], and its aberrant activation can contribute to cancer initiation and progression [75, 76]. In ESCC, BBOX1-AS1 activates the Wnt/β-catenin pathway by upregulating HOXB7 expression, enhancing the malignant behavior of ESCC cells, including cell proliferation, migration, and invasion, thereby promoting tumor progression [43].

BBOX1-AS1 has been linked to the MELK (Maternal Embryonic Leucine Zipper Kinase) and FAK (Focal Adhesion Kinase) signaling pathways. MELK and FAK are involved in cell proliferation, survival, and migration, making them critical players in cancer metastasis and resistance to therapies [77–81]. In NSCLC, the upregulation of BBOX1-AS1, induced by KLF5, acts as a miR-27a-5p sponge, leading to the activation of the MELK/FAK signaling pathway. This results in enhanced cell proliferation, migration, invasion, and EMT, promoting tumor progression [39].

## BBOX1-AS1 is involved in the drug resistance

Research has revealed that lncRNAs can contribute to sorafenib resistance, particularly in HCC [82, 83]. Sorafenib is a targeted therapy commonly used for treating advanced HCC, but the development of resistance to this drug remains a significant clinical challenge.

In the context of HCC [46], BBOX1-AS1 has been shown to promote tumor progression, autophagy, and drug resistance by upregulating a protein known as PHF8. The molecular mechanism involves BBOX1-AS1 enhancing the stability of PHF8 mRNA by targeting the PHF8 inhibitor miR-361-3p. By binding to miR-361-3p, BBOX1-AS1 effectively reduces the inhibitory effect of miR-361-3p on PHF8 expression, resulting in increased PHF8 levels in HCC cells. As a result of this regulatory axis, BBOX1-AS1 exerts its influence on various aspects of HCC pathogenesis. The elevated PHF8 expression, facilitated by BBOX1-AS1, contributes to tumor progression and increased autophagy levels in HCC cells. Moreover, the upregulation of PHF8 has been linked to the development of resistance to sorafenib treatment, making it a crucial factor in drug resistance mechanisms. This axis represents a promising target for therapeutic interventions aiming at overcoming drug resistance and enhancing the efficacy of sorafenib treatment in HCC patients. BBOX1-AS1’s involvement in the miR-361-3p/PHF8 axis is a key determinant of HCC progression and sorafenib resistance. Understanding the molecular mechanisms underlying this regulatory axis could provide valuable insights for the development of targeted therapies to address drug resistance and improve clinical outcomes for patients with HCC. Nevertheless, further investigation is warranted to explore the full potential of targeting this axis in HCC treatment strategies.

## Future perspectives and conclusion

**Fig. 8:**
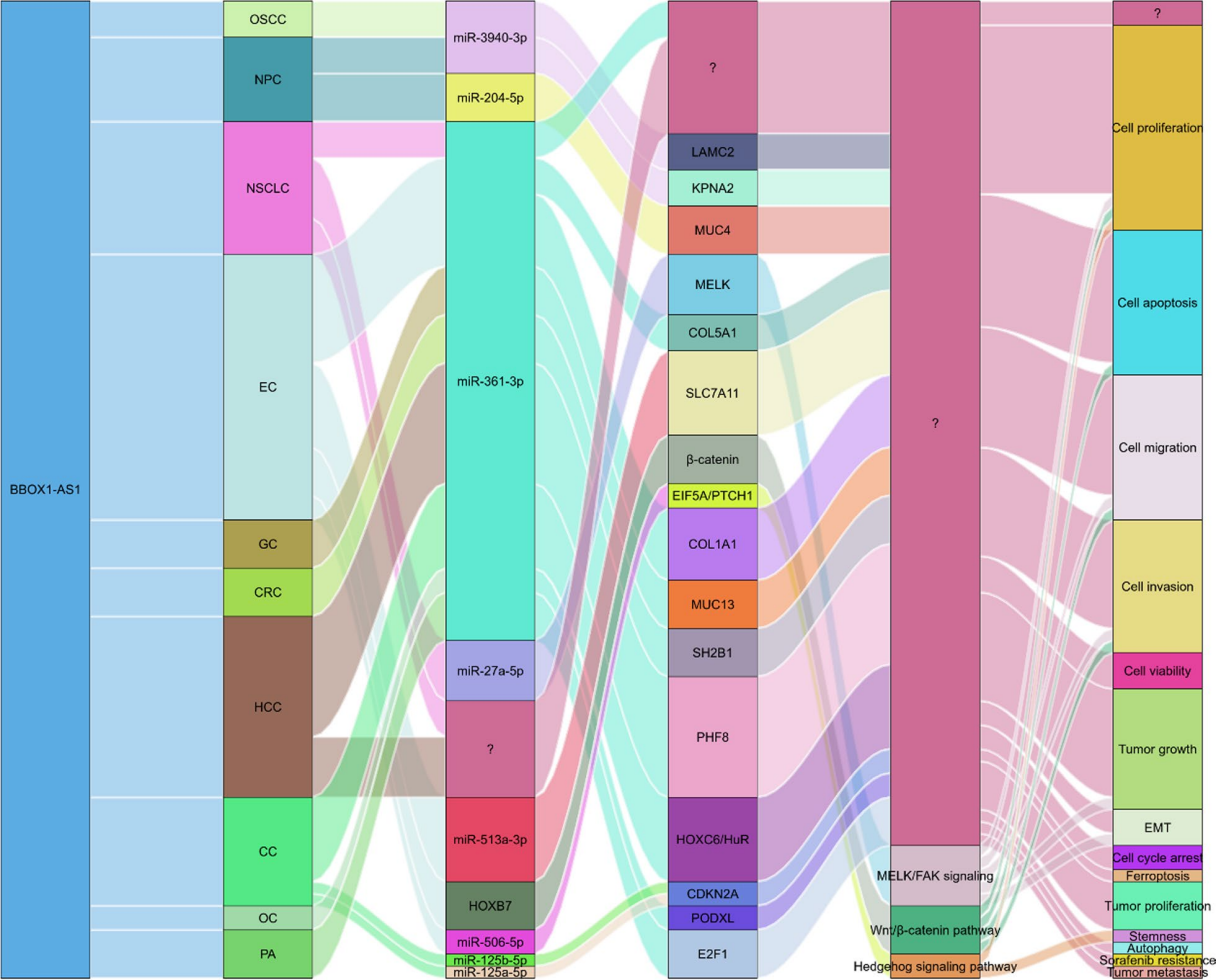
Summary of the role of lncRNA BBOX1-AS1 in regulating biological processes, related genes, and signaling pathways in tumor development

Numerous research teams have illuminated the regulatory role of BBOX1-AS1 in tumor development by influencing key molecules and genes associated with critical biological processes in tumorigenesis and disease progression (Fig. 8). These findings underscore the therapeutic potential of targeting BBOX1-AS1 in cancer treatment.

In recent years, BBOX1-AS1, an emerging lncRNA, has been found to be up-regulated in multiple of cancers. It shows potential as an oncogene and holds promise for tumor exploration and treatment. Importantly, high expression of BBOX1-AS1 is significantly correlated with various clinicopathological characteristics in these ten cancers, including tumor size, lymph node metastasis, distant metastasis, tumor differentiation, grade, and clinical stage. Moreover, up-regulation of BBOX1-AS1 is associated with poor prognosis in cancer patients [39–41, 43, 44, 46, 47, 53], and it displays diagnostic value in lung and esophageal carcinoma [38, 43].

BBOX1-AS1 establishes a complex ceRNA network by competitively binding 8 miRNAs and upregulating the expression of 15 target genes in 10 cancers. BBOX1-AS1 could also directly regulates downstream protein-coding gene HOXB7 [43] or acts as an mRNA stabilizer for PTCH1 mRNA [42] and HOXC6 mRNA [53]. BBOX1-AS1 has been implicated in the regulation of the Hedgehog signaling pathway [42], Wnt/β-catenin signaling pathway [43] and MELK/FAK signaling [39]. Moreover, BBOX1-AS1 increases tumor cell resistance to chemotherapeutic agents, including sorafenib resistance in hepatocellular carcinoma [46].

Despite the progress made, our understanding of BBOX1-AS1 is still limited, and its role in cancer warrants further exploration. Additional studies are required to assess BBOX1-AS1 expression in various types of solid and hematologic tumors, while evaluating its impact on overall survival and relapse-free periods in larger study populations. Comprehensive evaluation of BBOX1-AS1’s diagnostic potential across various tumor types is imperative. This should include analysis not only in clinical tissue samples but also in liquid biopsies, such as blood or other bodily fluids, to explore its clinical utility in early tumor diagnosis and monitoring. Furthermore, BBOX1-AS1 exhibits diverse functions in different types of cancers. While recent studies have portrayed it as a tumor promoter, it’s crucial to emphasize that the functional role of BBOX1-AS1 may be context-dependent. Its role can vary significantly based on the specific cellular or disease context, particularly in the case of hematologic malignancies and benign solid tumors, where its role remains largely unknown. The involvement of BBOX1-AS1 in tumorigenesis is complex, and a deeper comprehension of the precise mechanisms underlying BBOX1-AS1 in different tumor types is indispensable. It’s plausible that BBOX1-AS1 is involved in distinct pathways and has a more extensive ceRNA network. Furthermore, further research is warranted to unveil potential associations between BBOX1-AS1 and drug resistance.

In conclusion, BBOX1-AS1 shows promise as both a tumor marker and a potential therapeutic target in various cancers. It might be a valuable tool for clinical prognosis and diagnosis, offering potential opportunities for targeted interventions in cancer treatment. BBOX1-AS1 plays a significant role in the development and progression of tumors, influencing multiple oncogenic signaling pathways and promoting malignancy-related behaviors. These findings highlight the potential of BBOX1-AS1 as a predictive biomarker and an attractive target for cancer therapy. In the future, it is required to do more investigations of the regulatory mechanisms of BBOX1-AS1 in different cancer types and establish a more comprehensive BBOX1-AS1-based network to provide a theoretical foundation for targeted therapies involving BBOX1-AS1.

## Data Availability

Not applicable.

## References

[1] Gao N (2020). Long non-coding RNAs: the regulatory mechanisms, research strategies, and future directions in cancers. Front Oncol.

[2] Perkel JM (2013). Visiting noncodarnia. Biotechniques.

[3] Bánfai B (2012). Long noncoding RNAs are rarely translated in two human cell lines. Genome Res.

[4] Mattick JS (2023). Long non-coding RNAs: definitions, functions, challenges and recommendations. Nat Rev Mol Cell Biol.

[5] Li J, Xuan Z, Liu C (2013). Long non-coding RNAs and complex human diseases. Int J Mol Sci.

[6] Shi X (2013). Long non-coding RNAs: a new frontier in the study of human diseases. Cancer Lett.

[7] Zhao H (2023). Phytochemicals targeting lncRNAs: a novel direction for neuroprotection in neurological disorders. Biomed Pharmacother.

[8] Wu F (2023). Regulation mechanism and pathogenic role of lncRNA plasmacytoma variant translocation 1 (PVT1) in human diseases. Genes Dis.

[9] Khanmohammadi S, Fallahtafti P (2023). Long non-coding RNA as a novel biomarker and therapeutic target in aggressive B-cell non-hodgkin lymphoma: a systematic review. J Cell Mol Med.

[10] Kansara S (2023). The emerging regulatory roles of non-coding RNAs associated with glucose metabolism in breast cancer. Semin Cancer Biol.

[11] Huang Z (2023). Long non-coding RNA MAFG-AS1: a promising therapeutic target for human cancers. Biomed Pharmacother.

[12] Ghafouri-Fard S (2023). A review on the role of GHET1 in different cancers. Pathol Res Pract.

[13] Hung T, Chang HY (2010). Long noncoding RNA in genome regulation: prospects and mechanisms. RNA Biol.

[14] Mercer TR, Dinger ME, Mattick JS (2009). Long non-coding RNAs: insights into functions. Nat Rev Genet.

[15] Ghafouri-Fard S (2022). A review on the role of MCM3AP-AS1 in the carcinogenesis and tumor progression. Cancer Cell Int.

[16] Sanchez-Mejias A, Tay Y (2015). Competing endogenous RNA networks: tying the essential knots for cancer biology and therapeutics. J Hematol Oncol.

[17] Li R, Xu H, Gao X (2023). The ceRNA network regulates epithelial–mesenchymal transition in colorectal cancer. Heliyon.

[18] Li K, Yao T, Wang Z (2023). lncRNA-mediated ceRNA network in bladder cancer. Noncoding RNA Res.

[19] Liu Y (2022). Molecular mechanisms of thyroid cancer: a competing endogenous RNA (ceRNA) point of view. Biomed Pharmacother.

[20] Cesana M, Daley GQ (2013). Deciphering the rules of ceRNA networks. Proc Natl Acad Sci USA.

[21] Wu H (2023). LncRNA LZTS1-AS1 induces proliferation, metastasis and inhibits autophagy of pancreatic cancer cells through the miR-532 /TWIST1 signaling pathway. Cancer Cell Int.

[22] Li K (2023). HNRNPA2B1-mediated m(6)a modification of lncRNA MEG3 facilitates tumorigenesis and metastasis of non-small cell lung cancer by regulating miR-21-5p/PTEN axis. J Transl Med.

[23] Li J (2023). A novel pyroptosis-associated lncRNA LINC01133 promotes pancreatic adenocarcinoma development via miR-30b-5p/SIRT1 axis. Cell Oncol.

[24] Kan L, Yang M, Zhang H (2023). Long noncoding RNA PSMA3-AS1 functions as a competing endogenous RNA to promote gastric cancer progression by regulating the miR-329-3p/ALDOA axis. Biol Direct.

[25] Zhang Y (2023). LncRNA-BC069792 suppresses tumor progression by targeting KCNQ4 in breast cancer. Mol Cancer.

[26] Li D (2022). LINC02362 attenuates hepatocellular carcinoma progression through the miR-516b-5p/SOSC2 axis. Aging.

[27] Zhang S (2021). Long noncoding RNA Meg3 sponges miR-708 to inhibit intestinal tumorigenesis via SOCS3-repressed cancer stem cells growth. Cell Death Dis.

[28] Miao Y (2021). lncRNA GAS5, as a ceRNA, inhibits the proliferation of diffuse large B-cell lymphoma cells by regulating the miR-18a-5p/RUNX1 axis. Int J Oncol.

[29] Statello L (2021). Gene regulation by long non-coding RNAs and its biological functions. Nat Rev Mol Cell Biol.

[30] Zhang H (2013). Long non-coding RNA: a new player in cancer. J Hematol Oncol.

[31] Oo JA, Brandes RP, Leisegang MS (2022). Long non-coding RNAs: novel regulators of cellular physiology and function. Pflugers Arch.

[32] Li Y (2023). BBOX1-AS1 mediates trophoblast cells dysfunction via regulating hnRNPK/GADD45A axis. Biol Reprod.

[33] Yu Y (2022). Long non-coding RNA BBOX1 antisense RNA 1 increases the apoptosis of granulosa cells in premature ovarian failure by sponging miR-146b. Bioengineered.

[34] Wu H (2022). LncRNA BBOX1-AS1 promotes pituitary adenoma progression via sponging miR-361-3p/E2F1 axis. Anticancer Drugs.

[35] Zhao C, Shi W, Chen M (2022). Long non-coding RNA BBOX1-antisense RNA 1 enhances cell proliferation and migration and suppresses apoptosis in oral squamous cell carcinoma via the miR-3940-3p/laminin subunit gamma 2 axis. Bioengineered.

[36] Xiong J, Zhou L, Zhou Y (2023). LncRNA BBOX1-AS1 contributes to the development of nasopharyngeal carcinoma via miR-204-5p/MUC4 axis. Ann Clin Lab Sci.

[37] Jiang H, He Q, Liu T (2021). BBOX1-AS1 accelerates nasopharyngeal carcinoma progression by sponging mir-3940-3p and enhancing KPNA2 upregulation. Cancer Manag Res.

[38] Zhang Y (2022). Clinical significance and effect of lncRNA BBOX1-AS1 on the proliferation and migration of lung squamous cell carcinoma. Oncol Lett.

[39] Shi J (2021). KLF5-induced BBOX1-AS1 contributes to cell malignant phenotypes in non-small cell lung cancer via sponging miR-27a-5p to up-regulate MELK and activate FAK signaling pathway. J Exp Clin Cancer Res.

[40] Lian YF (2021). Targeted regulation of BBOX1-AS1 on mir-361-3p and its effect on the biological function of non-small cell lung cancer cell. J Biol Regul Homeost Agents.

[41] Ma R (2023). LncRNA BBOX1-AS1 targets miR-361-3p/COL1A1 axis to drive the progression of oesophageal carcinoma. Eur J Clin Invest.

[42] Hu L (2023). BBOX1-AS1 activates hedgehog signaling pathway to facilitate the proliferation and stemness of esophageal squamous cell carcinoma cells via miR-506-5p/EIF5A/PTCH1 axis. Curr Mol Pharmacol.

[43] Sheng J (2022). Long non-coding RNA BBOX1-AS1 exacerbates esophageal squamous cell carcinoma development by regulating HOXB7/β-catenin axis. Exp Cell Res.

[44] Pan C (2022). lncRNA BBOX1-AS1 silencing inhibits esophageal squamous cell cancer progression by promoting ferroptosis via miR-513a-3p/SLC7A11 axis. Eur J Pharmacol.

[45] Lu YH (2022). LncRNA BBOX1-AS1 contributes to the progression of esophageal carcinoma by targeting the miR-361-3p/COL5A1 axis. Biochem Genet.

[46] Tao H (2023). Oncogenic lncRNA BBOX1-AS1 promotes PHF8-mediated autophagy and elicits sorafenib resistance in hepatocellular carcinoma. Mol Ther Oncolytics.

[47] Tao H (2021). Construction of a ceRNA network and a prognostic lncRNA signature associated with vascular invasion in hepatocellular carcinoma based on weighted gene co-expression network analysis. J Cancer.

[48] Cai T (2022). Long noncoding RNA BBOX1-AS1 promotes the progression of gastric cancer by regulating the miR-361-3p/Mucin 13 signaling axis. Bioengineered.

[49] Shi ZL (2022). Identification of a prognostic colorectal cancer model including LncRNA FOXP4-AS1 and LncRNA BBOX1-AS1 based on bioinformatics analysis. Cancer Biother Radiopharm.

[50] Liu J (2022). BBOX1-AS1 contributes to colorectal cancer progression by sponging hsa-mir-361-3p and targeting SH2B1. FEBS Open Bio.

[51] Yao H (2021). LncRNA BBOX1-AS1 aggravates the development of ovarian cancer by sequestering mir-361-3p to augment PODXL expression. Reprod Sci.

[52] Wang T, Zhang XD, Hua KQ (2021). A ceRNA network of BBOX1-AS1-hsa-miR-125b-5pi>/hsa-miR-125a-5p-CDKN2A shows prognostic value in cervical cancer. Taiwan J Obstet Gynecol.

[53] Xu J (2020). LncRNA BBOX1-AS1 upregulates HOXC6 expression through mir-361-3p and HuR to drive cervical cancer progression. Cell Prolif.

[54] Kalluri R, Weinberg RA (2009). The basics of epithelial-mesenchymal transition. J Clin Invest.

[55] Huang Y, Hong W, Wei X (2022). The molecular mechanisms and therapeutic strategies of EMT in tumor progression and metastasis. J Hematol Oncol.

[56] Xu J (2022). The role of lncRNA-mediated ceRNA regulatory networks in pancreatic cancer. Cell Death Discov.

[57] Schwarzenbach H, Gahan PB (2023). Interplay between LncRNAs and microRNAs in breast cancer. Int J Mol Sci.

[58] Malakoti F (2023). Long noncoding RNA SNHG7-miRNA-mRNA axes crosstalk with oncogenic signaling pathways in human cancers. Chem Biol Drug Des.

[59] Tian Y (2023). Role of non-coding RNA intertwined with the Wnt/β-catenin signaling pathway in endometrial cancer (review). Mol Med Rep.

[60] Riquelme I (2023). Long non-coding RNAs (lncRNAs) as regulators of the PI3K/AKT/mTOR pathway in gastric carcinoma. Int J Mol Sci.

[61] Liu J, Ali MK, Mao Y (2023). Emerging role of long non-coding RNA MALAT1 related signaling pathways in the pathogenesis of lung disease. Front Cell Dev Biol.

[62] Hjazi A (2023). The cross-talk between LncRNAs and JAK-STAT signaling pathway in cancer. Pathol Res Pract.

[63] Ashrafizadeh M (2023). Noncoding RNAs as regulators of STAT3 pathway in gastrointestinal cancers: roles in cancer progression and therapeutic response. Med Res Rev.

[64] Zhang Y, Beachy PA (2023). Cellular and molecular mechanisms of hedgehog signalling. Nat Rev Mol Cell Biol.

[65] Shim S (2023). Calcium dynamics at the neural cell primary cilium regulate hedgehog signaling-dependent neurogenesis in the embryonic neural tube. Proc Natl Acad Sci USA.

[66] Zhou H (2022). Research progress on the hedgehog signalling pathway in regulating bone formation and homeostasis. Cell Prolif.

[67] Petrova R, Joyner AL (2014). Roles for hedgehog signaling in adult organ homeostasis and repair. Development.

[68] Sari IN (2018). Hedgehog signaling in cancer: a prospective therapeutic target for eradicating cancer stem cells. Cells.

[69] Gupta S, Takebe N, Lorusso P (2010). Targeting the hedgehog pathway in cancer. Ther Adv Med Oncol.

[70] Nairuz T (2023). Cancer stem cells: an insight into the development of metastatic tumors and therapy resistance. Stem Cell Rev Rep.

[71] Francescangeli F (2023). Dormancy, stemness, and therapy resistance: interconnected players in cancer evolution. Cancer Metastasis Rev.

[72] Dianat-Moghadam H (2023). Engaging stemness improves cancer immunotherapy. Cancer Lett.

[73] Komiya Y, Habas R (2008). Wnt signal transduction pathways. Organogenesis.

[74] Steinhart Z, Angers S (2018). Wnt signaling in development and tissue homeostasis. Development.

[75] Zhan T, Rindtorff N, Boutros M (2017). Wnt signaling in cancer. Oncogene.

[76] Chen Y (2021). The involvement of noncanonical wnt signaling in cancers. Biomed Pharmacother.

[77] Ganguly R (2015). MELK-a conserved kinase: functions, signaling, cancer, and controversy. Clin Transl Med.

[78] Tang Q (2020). MELK is an oncogenic kinase essential for metastasis, mitotic progression, and programmed death in lung carcinoma. Signal Transduct Target Ther.

[79] Chuang HH (2022). FAK in cancer: from mechanisms to therapeutic strategies. Int J Mol Sci.

[80] Sulzmaier FJ, Jean C, Schlaepfer DD (2014). FAK in cancer: mechanistic findings and clinical applications. Nat Rev Cancer.

[81] Lee BY (2015). FAK signaling in human cancer as a target for therapeutics. Pharmacol Ther.

[82] Hu X (2021). The role of non-coding RNAs in the sorafenib resistance of hepatocellular carcinoma. Front Oncol.

[83] Wei L (2019). The emerging role of microRNAs and long noncoding RNAs in drug resistance of hepatocellular carcinoma. Mol Cancer.

